# A taxonomic revision of the Neoserica (sensu lato) calva group (Coleoptera, Scarabaeidae, Sericini)

**DOI:** 10.3897/zookeys.448.8368

**Published:** 2014-10-20

**Authors:** Wan-Gang Liu, Silvia Fabrizi, Ming Bai, Xing-Ke Yang, Dirk Ahrens

**Affiliations:** 1 Key Laboratory of Zoological Systematics and Evolution, Institute of Zoology, Chinese Academy of Sciences, Box 92, No. 1, Beichen West Road, Chaoyang District, Beijing, 100101, P.R. China; 2 University of Chinese Academy of Sciences, Yuquan Road, Shijingshan, Beijing, 100039, P.R. China; 3 Zoologisches Forschungsmuseum A. Koenig, Adenauerallee 160, 53113 Bonn, Germany

**Keywords:** Beetles, chafers, *Neoserica*, China, South Korea, new species

## Abstract

The species of the Neoserica (sensu lato) calva group are revised. *Neoserica calva* Frey, 1972, **comb. n.** is redescribed. Thirteen new species are described from China and South Korea: *Neoserica ailaoshanica* **sp. n.**, *Neoserica anonyma* **sp. n.**, *Neoserica calvoides* **sp. n.**, *Neoserica gulinqingensis* **sp. n.**, *Neoserica koelkebecki* **sp. n.**, *Neoserica liangi* **sp. n.**, *Neoserica luxiensis* **sp. n.**, *Neoserica menghaiensis* **sp. n.**, *Neoserica mengi* **sp. n.**, *Neoserica taipingensis* **sp. n.**, *Neoserica zheijangensis* **sp. n.**, *Neoserica zhibenshanica* **sp. n.**, and *Neoserica zongyuani* **sp. n.** A key to Sericini genera with multilamellate antenna and species groups of *Neoserica* of mainland Asia as well as a key to species of the *Neoserica calva* group are provided. A map of species distribution is given, habitus and male genitalia are illustrated.

## Introduction

In the present study we improve the taxonomy of the representatives of *Neoserica* Brenske, 1894, related to *Neoserica calva* Frey, 1972, described originally from Fujian (China). According to our present knowledge the species group is restricted to southern China and South Korea. The species of this group are characterised by the bidentate protibia, antennal club composed of four or five antennomeres in male, frons being shiny anteriorly and dull and finely setose in posterior third or half, and posterior margin of metafemur being serrated ventrally and dorsally. Here, thirteen new species are described from Southern China or South Korea.

## Material and methods

The terminology and methods used for measurements, specimen dissection and genital preparation follow Ahrens (2004). Data from specimens examined are cited in the text with original label contents given in quotation marks, multiple labels are separated by a “/”. Male genitalia were glued to a small pointed card attached to the specimen. Descriptions and illustrations of new taxa are based on the holotype specimen, while the variation of other specimens is described separately. All descriptions and measurements were made under an Olympus SZX 12 microscope, and all genital and habitus illustrations were made with a digital camera (AxioCam HRc) attached to a stereo microscope (Zeiss Stereo Discovery V20) and Axio Version 4.8 software. Measurements refer to the maximum extension of the specimen or the named structure. The distribution map was generated using Q-GIS 2.0.1 and Adobe Photoshop CS4.

Type specimens and other examined material are deposited in the following institutions:

BMH Bishop Museum Honululu, Hawai, U.S.A.;

CPPB Coll. Petr Pacholatko, Brno, Czech Republic;

HBUM Museum of Hebei University, Baoding (Hebei Prov.) China;

IZAS Institute of Zoology, Chinese Academy of Sciences, Beijing, China;

LSSYU College of Life Sciences, Sun Yat-sen University, Guangzhou (Guangdong Prov.), China;

MHNG Museum d’Histoir Naturelle de Geneve, Switzerland;

NMPC National Museum Prague (Natural History), Czech Republic;

ZFMK Zoologisches Forschungsmuseum A. Koenig, Bonn, Germany.

### A preliminary key to the species groups and genera of Chinese Sericini with a multilamellate antennal club

**Table:** 

1	Hypomeron not carinate	***Tetraserica* Ahrens, 2004**
1’	Hypomeron carinate	**2**
2	Antennal club in female composed of 3 antennomeres	**3**
2’	Antennal club in female composed of more than 3 antennomeres	**15**
3	Posterior margin of metafemur serrate ventrally and dorsally	**4**
3’	Posterior margin of metafemur smooth ventrally	**7**
4	Anterior angles of pronotum obsolete	**5**
4’	Anterior angles of pronotum acute and moderately produced	**Neoserica (s. l.) calva group**
5	Dorsal surface nearly glabrous	***Gastroserica* Brenske, 1897**
5’	Dorsal surface densely setose	**6**
6	Metatibia beside dorsal margin with a serrated longitudinal line or carina	***Neoserica* (s.str.) Brenske, 1894**
6’	Metatibia beside dorsal margin without a serrated longitudinal line or carina	***Calloserica* Brenske, 1894**
7	Metatibia beside dorsal margin with a serrated longitudinal line or carina	***Lasioserica* Brenske, 1896**
7’	Metatibia beside dorsal margin without a serrated longitudinal line or carina	**8**
8	Antennal club in males long and reflexed	***Anomalophylla* Reitter, 1887**
8’	Antennal club in males short or moderately long and straight	**9**
9	Protibia bidentate	**10**
9’	Protibia tridentate	***Trioserica* Moser, 1922**
10	Elytra bicolored, yellowish or reddish brown and black	**11**
10’	Elytra unicolored	**12**
11	Parameres symmetrical	***Oxyserica* Brenske, 1900**
11’	Parameres asymmetrical	***Microserica* Brenske, 1894**
12	Apex of metatibia shallowly truncate at interior apex near tarsal articulation	**13**
12’	Apex of metatibia sharply truncate at interior apex near tarsal articulation	**14**
13	Dorsal surface yellowish brown to reddish brown, strongly and simply shiny	**Neoserica (s. l.) lubrica group**
13’	Dorsal surface dull or iridescent shiny	**Neoserica (s. l.) vulpes group, other *Neoserica* (s.l.)**
14	Pronotum and elytra always nearly glabrous	***Sericania* Motschulsky, 1860** (see also couplet 21)
14’	Pronotum and elytra always distinctly setose	***Leuroserica* Arrow, 1946, *Gynaecoserica* Brenske, 1896**
15	Labrum without a transverse rim of very dense, short and robust setae	**16**
15’	Labrum short, with a transverse rim of very dense, short and robust setae. Dorsal surface densely setose	**Neoserica (s. l.) pilosula group**
16	Metatibia slender and long	**17**
16’	Metatibia short and wide	**Neoserica (s. l.) uniformis group** & **Neoserica (s. l.) multifoliata group** (from Indochina)
17	Antennal club of males with 7 antennomeres	**18**
17’	Antennal club of males with 7, 6 or less antennomeres	**19**
18	Metafemur with a continuously serrated line adjacent to the anterior margin of metafemur. Protibia more or less distinctly tridentate	**Neoserica (s. l.) septemlamellata group**
18’	Metafemur without a continuously serrated line adjacent to the anterior margin of metafemur. Protibia always distinctly bidentate	***Nepaloserica* Frey, 1965**
19	Basis of labroclypeus dull. Antennal club of males with 6 antennomeres	**20**
19’	Antennal club of males with 5 or 4 antennomeres	**21**
20	Angle between basis of hypomeron and that of pronotum strongly rounded, angle between surfaces of hypomeron and pronotum basally blunt. Hypomeron basally strongly produced ventrally and transversely sulcate	***Lepidoserica* Nikolaev, 1979**
20’	Angle between basis of hypomeron and that of pronotum sharp, angle between surfaces of hypomeron and pronotum shar. Hypomeron basally not produced ventrally and not sulcate	**Neoserica (s. l.) abnormis group**
21	Apex of metatibia shallowly truncate at interior apex near tarsal articulation	**22**
21’	Apex of metatibia deeply truncate at interior apex near tarsal articulation	***Sericania* Motschulsky, 1860** (see also couplet 14)
22	Body surface strongly shiny. Body smaller (5.7–6.6 mm)	**Neoserica (s. l.) speciosa group**
22’	Body surface dull. Body larger (8 mm)	***Chrysoserica* Brenske, 1897**

### Key to species of the *Neoserica calva* group (♂♂):

**Table:** 

1	Eyes small: ratio diameter/interocular distance < 0.65	**2**
1’	Eyes larger: ratio diameter/interocular distance > 0.72	**7**
2	Antennal club longer, 3 times as long as remaining antennomeres combined	***Neoserica zhibenshanica* sp. n.**
2’	Antennal club shorter, at maximum 1.7 times as long as remaining antennomeres combined	**3**
3	Antennal club short, at maximum 1.2 times as long as remaining antennomeres combined. Phallobase without apical process	**4**
3’	Antennal club longer, 1.4 to 1.7 times as long as remaining antennomeres combined. Phallobase with apical process	**5**
4	Metatibia moderately wide, ratio width/length: 1/3.3. Left paramere not reduced in length	***Neoserica anonyma* sp. n.**
4’	Metatibia more stout, ratio width/length: 1/2.8. Left paramere strongly reduced in length	***Neoserica mengi* sp. n.**
5	Phallobase with narrow dorsal process. Species from South Korea	***Neoserica koelkebecki* sp. n.**
5’	Phallobase with wide dorsolateral process	**6**
6	Right paramere more elongate, narrow	***Neoserica ailaoshanica* sp. n.**
6’	Right paramere shorter, dorsoventrally strongly widened at middle	***Neoserica luxiensis* sp. n.**
7	Antennal club moderately long, at maximum 1.4 times as long as remaining antennomeres combined	**8**
7’	Antennal club long, at least twice as long as remaining antennomeres combined	**11**
8	Eyes very large, ratio diameter/interocular distance > 0.9. Metatibia in basal half with blunt carina beside dorsal margin bearing a few short robust setae in punctures with serrated margin	**9**
8’	Eyes smaller, ratio diameter/interocular distance < 0.8. Metatibia in basal half without a blunt carina beside dorsal margin	**10**
9	Left paramere reduced in length, not visible under the largely widened dorsal lobe of right paramere	***Neoserica calvoides* sp. n.**
9’	Left paramere not reduced in length, subequal in length to the less widened dorsal lobe of right paramere	***Neoserica gulinqingensis* sp. n.**
10	Phallobase distinctly widened at apex. Left paramere straight at apex	***Neoserica liangi* sp. n.**
10’	Phallobase not widened at apex. Left paramere hooked at apex	***Neoserica napoana* sp. n.**
11	Antennal club composed of 5 antennomeres	***Neoserica zheijangensis* sp. n.**
11’	Antennal club composed of 4 antennomeres	**12**
12	Eyes very large, ratio diameter/interocular distance > 1.0	***Neoserica calva* Frey**
12’	Eyes smaller, ratio diameter/interocular distance < 0.85	**13**
13	Legs moderately long, ratio metatibial width/length: 1/3.4	***Neoserica taipingensis* sp. n.**
13’	Legs longer, ratio metatibial width/length: 1/3.9	***Neoserica zongyuani* sp. n.**

## Systematics

### Neoserica (s. l.) calva

Taxon classificationAnimaliaColeopteraScarabaeidae

(Frey, 1972) comb. n.

Figs 1A–D , 6

Trichoserica calva Frey, 1972: 173.
Serica calva : Ahrens 2006: 245, 2007: 36.

#### Type material examined.

Holotype: ♂ “Kuatun 2300 m 27,40 n. Br. 117,40 ö. L. J. Klapperich 18.4.1938 (Fukien)/ Ophthalmoserica Type clava G. Frey 1972/ Trichoserica calva” (ZFMK). Paratypes: 1 ♂ “Kuatun 2300 m 27,40 n. Br. 117,40 ö. L. J. Klapperich 28.4.1938 (Fukien)/ Ophthalmoserica Paratype clava G. Frey 1972” (ZFMK), 1 ♂ “Kuatun 2300 m 27,40 n. Br. 117,40 ö. L. J. Klapperich 15.4.1938 (Fukien)/ Ophthalmoserica Paratype clava G. Frey 1972” (ZFMK).

#### Additional material examined.

1 ex. “Chine 31.IV.46 Kuatun, Fukien leg. Tschung-Sen” (MHNG), 1 ex. “Chine 8.IV.46 Kuatun, Fukien leg. Tschung-Sen” (MHNG), 1 ex. “Chine 22.VII.46 Kuatun, Fukien leg. Tschung-Sen” (MHNG), 33 ex. “China: Hunan; Mupu Mt. 1600 m, Pingjiang VIII-2003, leg. Li et al.” (ZFMK), 924 ex. “China: Hunan: Jiucai Ling 25°32'N, 111°22'E, ~300m, iv.2006, leg. V. Siniaev” (ZFMK), 1 ex. “Kuatun 2300 m 27,40 n. Br. 117,40 ö. L. J. Klapperich 31.5.1938 (Fukien)/ ex. Coll. V. Balthasar Natinal Museum Prague, Czech Republic” (NMPC), 1 ex. “Kuatun 2300 m 27,40 n. Br. 117,40 ö. L. J. Klapperich 17.5.1938 (Fukien)/ ex. Coll. V. Balthasar National Museum Prague, Czech Republic” (NMPC), 1 ex. “Kuatun 2300 m 27,40 n. Br. 117,40 ö. L. J. Klapperich 21.4.1938 (Fukien)/ ex. Coll. V. Balthasar National Museum Prague, Czech Republic” (NMPC), 1 ♂ “Aotou, Huangkeng, Jianyang, Fujian, 2.V.1960, 950m, leg. Zhang Yinran” (IZAS), 1 ♂ “San’gang, Fujian, 17.IV.1981, leg. Wang Jiashe” (IZAS).

#### Redescription.

Body length: 6.1 mm, length of elytra: 4.7 mm, width: 3.6 mm. Body oblong, dark reddish brown, antennal club yellowish brown, dorsal surface dull and nearly glabrous, labroclypeus and anterior two thirds of frons shiny.

Labroclypeus subrectangular, only little wider than long, widest at middle; lateral margins convex and moderately convergent anteriorly and posteriorly; anterior angles moderately rounded; anterior margin moderately sinuate medially; margins strongly reflexed; surface weakly elevated medially and shiny, finely and very densely punctate, with a few single setae. Frontoclypeal suture distinctly incised, weakly elevated and moderately angled medially. Smooth area anterior to eye approximately twice as wide as long. Ocular canthus moderately long and narrow, finely and sparsely punctate, with a terminal seta. Frons on posterior third dull, finely and densely punctate, midline impunctate and slightly elevated; with a few erect setae beside eyes. Eyes very large, ratio diameter/interocular width: 1.1. Antenna with ten antennomeres, club with four antennomeres and strongly reflexed, 2.3 times as long as remaining antennomeres combined. Mentum elevated and slightly flattened anteriorly. Labrum transverse, short, not produced medially, with weak median sinuation.

Pronotum short and transverse, almost twice as wide as long, widest at base; lateral margins nearly straight and subparallel in basal half, moderately convex and strongly convergent anteriorly; anterior angles weakly produced and blunt, slightly rounded at tip; posterior angles nearly right-angled and moderately rounded at tip; anterior margin with a fine and complete marginal line, weakly convexly produced medially; surface densely and finely punctate, with minute setae in punctures; lateral border sparsely setose, anterior one glabrous; hypomeron distinctly carinate basally. Scutellum long, triangular, with fine, very dense punctures, glabrous, punctures less dense on basal midline.

Elytra oblong, widest in posterior third; striae weakly impressed, finely and moderately densely punctate; even intervals flat, with evenly and moderately dense punctures; odd intervals convex, with sparse, fine punctures concentrated along striae, impunctate medially, with minute setae in punctures. Epipleural edge fine, ending at moderately curved external apical angle of elytra; epipleura densely setose; apical border with a fine rim of microtrichomes (visible at 100× magnification).

Ventral surface dull, finely and densely punctate. Metasternum except long seta on disc nearly glabrous, sparsely covered with minute setae in punctures. Metacoxa glabrous, with a few single setae laterally. Abdominal sternites finely and densely punctuate, glabrous except minute setae in punctures, with a transverse row of coarse punctures each bearing a robust long seta. Mesosternum between mesocoxae as wide as mesofemur. Ratio of length of metepisternum/metacoxa: 1/1.23. Pygidium weakly convex and dull, coarsely and densely punctate, without smooth midline, with a few long setae at apex, otherwise glabrous.

Legs slender. Femora with two longitudinal rows of setae, finely and sparsely punctate. Metafemur moderately shiny and sparsely finely punctate; anterior margin acute, behind anterior margin without serrated line; posterior margin in apical half serrated ventrally and moderately widened at apex; posterior margin finely serrated dorsally, glabrous. Metatibia slender and moderately long, widest at apex, ratio of width/length: 1/3.88; dorsal margin sharply carinate, with two groups of spines; basal group at middle, apical group at three quarters of metatibial length; basally with a few short robust setae in single robust punctures; external face longitudinally convex, finely and sparsely punctate; ventral margin finely serrated, with three robust setae, with the apical one being more distant; medial face impunctate, glabrous, apex sharply truncate interiorly near tarsal articulation. Tarsomeres ventrally with sparse, short setae, not carinate laterally, with fine sparse punctures dorsally; metatarsomeres with a strongly serrated ventral ridge; metatarsomere I as long as following two tarsomeres combined and half of its length longer than dorsal tibial spur. Protibia moderately long, bidentate, bluntly widened laterally before basal tooth; anterior claws symmetrical, basal tooth of inner claw sharply truncate at apex.

**Figure 1.**
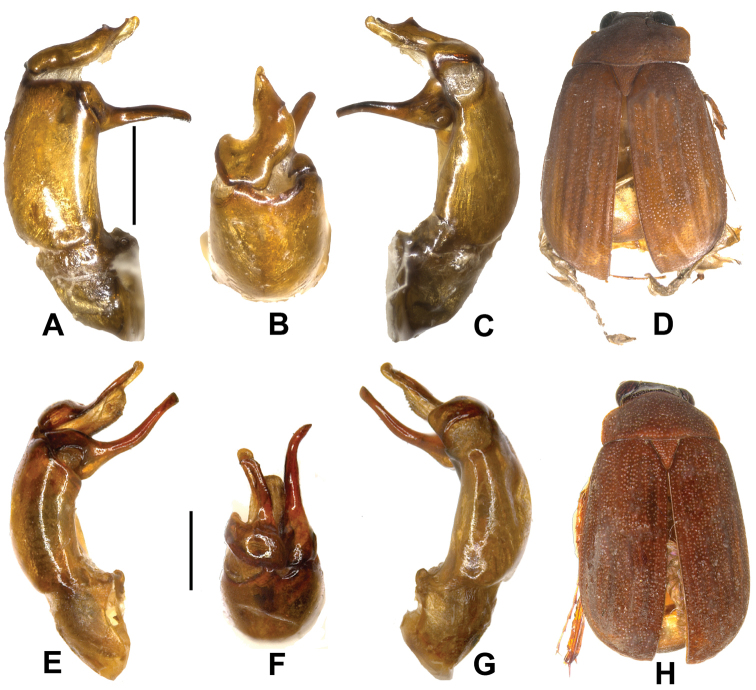
**A–D** *Neoserica calva* (Frey) (lectotype) **E–H** *Neoserica zongyuani* sp. n. (holotype). **A, E** aedeagus, left side lateral view **C, G** aedeagus, right side lateral view **B, F** parameres, dorsal view **D, H:** habitus. Scale: 0.5 mm. Habitus not to scale.

Aedeagus. Fig. 1A–C.

### Neoserica (s. l.) zongyuani sp. n.

Taxon classificationAnimaliaColeopteraScarabaeidae

http://zoobank.org/5243A034-45CB-44B7-8C93-373ED01BE211

Figs 1E–H , 6

#### Type material examined.

Holotype: ♂ [China] “Qingyin’ge, Mts. Emeishan, Sichuan, 20.IV.1957, 800–1000m, leg. Wang Zongyuan” (IZAS). Paratypes: 1 ♂ [China] “Guanyinge, Mts. Emeishan, Sichuan, 24.IV.1957, 800-1000m, leg. Wang Zongyuan” (IZAS), 1 ♂ [China] “Baoguosi Temple, Mts. Emeishan, Sichuan, 22.IV.1957, 550–750m, leg. Lu Youcai” (IZAS), 1 ♂ [China] “Qingyin’ge, Mts. Emeishan, Sichuan, 16.V.1957, 800–1000m, leg. Zhu Fuxing” (ZFMK).

#### Description.

Body length: 6.0 mm, length of elytra: 4.4 mm, width: 3.3 mm. Body oblong, dark reddish brown, antennal club yellowish brown, dorsal surface dull and nearly glabrous, labroclypeus and anterior two thirds of frons shiny.

Labroclypeus subrectangular, only little wider than long, widest at base; lateral margins in basal half subparallel and straight, strongly convergent and convex anteriorly; anterior angles moderately rounded; anterior margin moderately sinuate medially; margins moderately reflexed; surface weakly elevated medially and shiny, finely and very densely punctate, with a few single setae. Frontoclypeal suture distinctly incised, weakly elevated and moderately angled medially. Smooth area anterior to eye approximately twice as wide as long. Ocular canthus moderately long and narrow, finely and sparsely punctate, with a single terminal seta. Frons on posterior third dull, finely and densely punctate, anterior midline impunctate and slightly elevated; with a few erect setae beside eyes. Eyes large, ratio diameter/interocular width: 0.83. Antenna with ten antennomeres, club with four antennomeres and strongly reflexed, 2.3 times as long as remaining antennomeres combined. Mentum elevated and slightly flattened anteriorly. Labrum transverse, short, not produced medially, with weak median sinuation.

Pronotum moderately transverse, almost twice as wide as long, widest at base; lateral margins nearly straight and weakly convergent in basal half, moderately convex and moderately convergent anteriorly; anterior angles weakly produced and blunt; posterior angles nearly right-angled and moderately rounded at tip; anterior margin with a fine and complete marginal line, weakly convexly produced medially; surface densely and finely punctate, with minute setae in punctures; lateral and anterior border sparsely setose; hypomeron distinctly carinate basally. Scutellum long, with fine, very dense punctures, glabrous.

Elytra oblong, widest in posterior third; striae weakly impressed, finely and moderately densely punctate; even intervals flat, with evenly and moderately dense punctures; odd intervals convex, with sparse, fine punctures concentrated along striae, impunctate medially, with minute setae in punctures. Epipleural edge fine, ending at moderately curved external apical angle of elytra; epipleura densely setose; apical border with a fine rim of microtrichomes (visible at 100× magnification).

Ventral surface dull, finely and densely punctate. Metasternum except long seta on disc nearly glabrous, sparsely covered with minute setae in punctures. Metacoxa glabrous, with a few single setae laterally. Abdominal sternites finely and densely punctuate, glabrous except minute setae in punctures, with a transverse row of coarse punctures each bearing a robust long seta. Mesosternum between mesocoxae as wide as mesofemur. Ratio of length of metepisternum/metacoxa: 1/1.24. Pygidium moderately convex and dull, coarsely and densely punctate, with narrow smooth midline, with a few long setae at apex, otherwise glabrous.

Legs slender. Femora with two longitudinal rows of setae, finely and sparsely punctate. Metafemur moderately shiny and sparsely finely punctate; anterior margin acute, behind anterior margin without serrated line; posterior margin in apical half serrated ventrally and moderately widened at apex; posterior margin finely serrated dorsally, glabrous. Metatibia slender and moderately long, widest at apex, ratio of width/length: 1/3.9; dorsal margin sharply carinate, with two groups of spines; basal group at middle, apical group at three quarters of metatibial length; in basal half with a few short robust setae in single robust punctures with serrated margin; external face longitudinally convex, finely and sparsely punctate; ventral margin finely serrated, with three robust setae, with the apical one being more distant; medial face impunctate, glabrous, apex sharply truncate interiorly near tarsal articulation. Tarsomeres ventrally with sparse, short setae, not carinate laterally, with fine sparse punctures dorsally; metatarsomeres missing in holotype. Protibia moderately long, bidentate, bluntly widened laterally before basal tooth; anterior claws symmetrical, basal tooth of inner claw sharply truncate at apex.

Aedeagus. Fig. 1E–G.

Female unknown.

#### Diagnosis.

*Neoserica zongyuani* sp. n. differs form *Neoserica calva* by the slightly smaller eyes and the shape of the parameres: the left paramere is longer than that in *Neoserica calva*, the right paramere is narrowed abruptly behind base.

#### Etymology.

The new species is named after one of the collectors of the type series, Wang Zongyuan.

#### Variation.

Length: 6.0 mm, length of elytra: 4.4–4.6 mm, width: 3.3–3.6 mm.

### Neoserica (s. l.) menghaiensis sp. n.

Taxon classificationAnimaliaColeopteraScarabaeidae

http://zoobank.org/31693723-E6C2-4160-B0F4-B42A861C2C65

Figs 2A–D , 6

#### Type material examined.

Holotype: ♂ [China] “Menghai, Xishuangbanna, Yunnan, 18.VII.1958, 1200-1600m, leg. Wang Shuyong” (IZAS). Paratype: 1 ♂ “Defu, Napo, Guangxi, 19.VI.2000, 1350m, leg. Li Wenzhu” (ZFMK).

#### Description.

Body length: 5.1 mm, length of elytra: 3.9 mm, width: 3.5 mm. Body oval, dark reddish brown, antennal club yellowish brown, dorsal surface dull and nearly glabrous, labroclypeus and anterior half of frons shiny.

Labroclypeus subtrapezoidal, distinctly wider than long, widest at base; lateral margins strongly convergent and convex anteriorly; anterior angles blunt; anterior margin distinctly sinuate medially; margins moderately reflexed; surface weakly elevated medially and shiny, finely and densely punctate, with a few single setae. Frontoclypeal suture indistinctly incised, weakly elevated and moderately angled medially. Smooth area anterior to eye approximately twice as wide as long. Ocular canthus short and narrow, finely and sparsely punctate, with a single terminal seta. Frons on posterior half dull, finely and densely punctate, anterior midline narrowly impunctate and not elevated; with a few erect setae beside eyes and dense fine setae on posterior half. Eyes large, ratio diameter/interocular width: 0.72. Antenna with ten antennomeres, club with four antennomeres and straight, 1.2 times as long as remaining antennomeres combined. Mentum elevated and slightly flattened anteriorly. Labrum transverse, short, not produced medially, with weak median sinuation.

Pronotum moderately transverse, almost twice as wide as long, widest at base; lateral margins weakly evenly convex and weakly convergent, more strongly convergent in anterior third; anterior angles distinctly produced and sharp; posterior angles strongly rounded; anterior margin with a fine and complete marginal line, weakly convexly produced medially; surface densely and finely punctate, with minute setae in punctures; lateral and anterior border sparsely setose; hypomeron distinctly carinate basally. Scutellum long, with fine, dense punctures, at base punctures less dense, glabrous.

Elytra short-oval, widest in posterior third; striae weakly impressed, finely and moderately densely punctate; even intervals flat, with evenly and moderately dense punctures; odd intervals convex, with sparse, fine punctures concentrated along striae, impunctate medially, with minute setae in punctures. Epipleural edge fine, ending at moderately curved external apical angle of elytra; epipleura densely setose; apical border with a fine rim of microtrichomes (visible at 100× magnification).

Ventral surface dull, finely and densely punctate. Metasternum except long seta on disc nearly glabrous, sparsely covered with minute setae in punctures. Metacoxa glabrous, with a few single setae laterally. Abdominal sternites finely and densely punctuate, glabrous except minute setae in punctures, with a transverse row of coarse punctures each bearing a robust long seta. Mesosternum between mesocoxae as wide as mesofemur. Ratio of length of metepisternum/metacoxa: 1/1.39. Pygidium weakly convex and dull, coarsely and densely punctate, with narrow smooth midline, with a few long setae at apex, otherwise glabrous.

Legs moderately slender. Femora with two longitudinal rows of setae, finely and sparsely punctate. Metafemur moderately shiny and sparsely finely punctate; anterior margin acute, behind anterior margin without serrated line; posterior margin entirely serrated ventrally and moderately widened at apex; posterior margin finely serrated dorsally, glabrous. Metatibia slender and moderately long, widest at apex, ratio of width/length: 1/3.2; dorsal margin indistinctly carinate, with two groups of spines; basal group at middle, apical group at three quarters of metatibial length; in basal half with a few short robust setae in single robust punctures with serrated margin; external face longitudinally convex, finely and sparsely punctate; ventral margin finely serrated, with three robust setae, with the apical one being more distant; medial face impunctate, glabrous, apex shallowly sinuate interiorly near tarsal articulation. Tarsomeres ventrally with sparse, short setae, not carinate laterally, with fine sparse punctures dorsally; metatarsomeres with a strongly serrated ventral ridge; metatarsomere I as long as following two tarsomeres combined and half of its length longer than dorsal tibial spur. Protibia short, bidentate, not widened laterally before basal tooth; anterior claws symmetrical, basal tooth of inner claw sharply truncate at apex.

**Figure 2.**
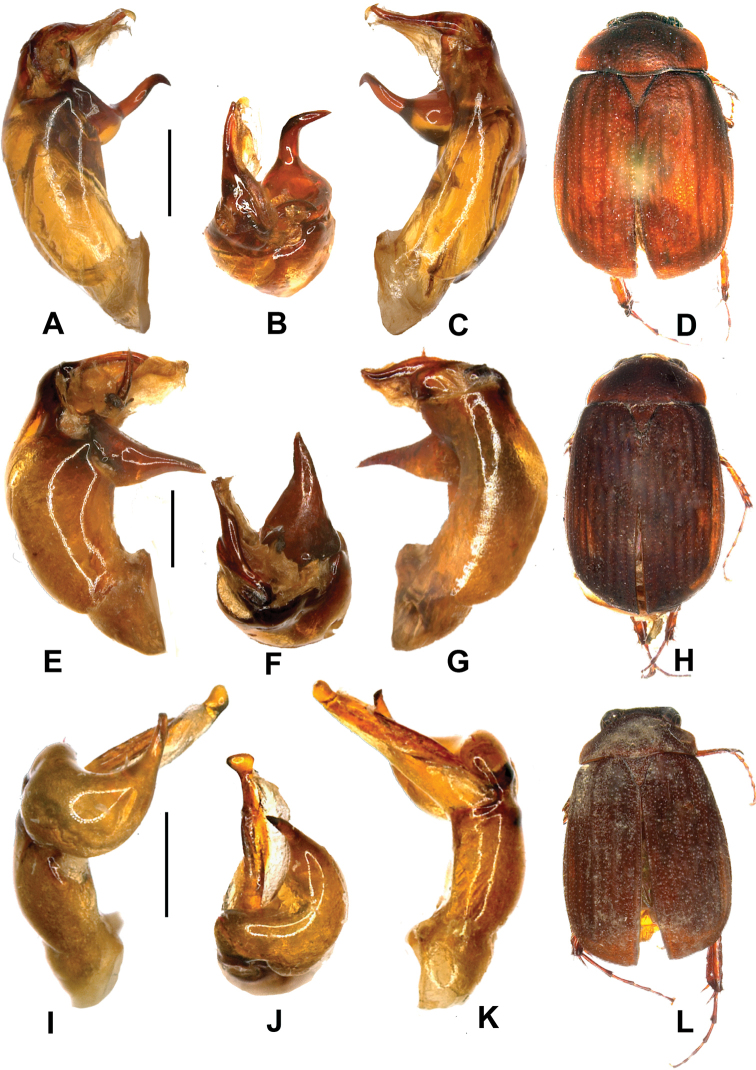
**A–D** *Neoserica menghaiensis* sp. n. (holotype) **E–H** *Neoserica liangi* sp. n. (holotype) **I–L** *Neoserica calvoides* sp. n. (holotype). **A, E, I** aedeagus, left side lateral view **C, G, K** aedeagus, right side lateral view **B, F, J** parameres, dorsal view **D, H, L** habitus. Scale: 0.5 mm. Habitus not to scale.

Aedeagus. Fig. 2A–C.

Female unknown.

#### Diagnosis.

*Neoserica menghaiensis* sp. n. differs form *Neoserica calva* by the more wider body shape, shorter and straight antennal club, smaller eyes, less sharply carinate dorsal margin of metatibia, and the shape of the parameres: the left paramere is much more widened at base than in that *Neoserica calva*, the right paramere is almost straight.

#### Etymology.

The new species is named after its type locality, Menghai.

#### Variation.

Body length of the paratypes: 5.1–5.7 mm, length of elytra: 3.9–4.3 mm.

### Neoserica (s. l.) liangi sp. n.

Taxon classificationAnimaliaColeopteraScarabaeidae

http://zoobank.org/ED2AE588-3116-48CB-9D73-B01846F2F4D7

Figs 2E–H , 6

#### Type material examined.

Holotype: ♂ [China] “Caiyanghe Nature Reserve, Pu’er, Yunnan, 28–29.VII.2007, leg. Liang Geqiu” (LSSYU).

#### Description.

Body length: 5.9 mm, length of elytra: 4.5 mm, width: 3.6 mm. Body oval, dark reddish brown, antennal club yellowish brown, dorsal surface dull and nearly glabrous, labroclypeus and anterior half of frons shiny.

Labroclypeus subtrapezoidal, distinctly wider than long, widest at base; lateral margins strongly convergent and convex anteriorly; anterior angles blunt; anterior margin distinctly sinuate medially; margins moderately reflexed; surface weakly elevated medially and shiny, finely and densely punctate, with a few single setae. Frontoclypeal suture indistinctly incised, weakly elevated and moderately angled medially. Smooth area anterior to eye approximately twice as wide as long. Ocular canthus short and narrow, finely and sparsely punctate, with a single terminal seta. Frons on posterior half dull; finely and densely punctate; with a few erect setae beside eyes and dense fine setae on posterior half. Eyes large, ratio diameter/interocular width: 0.75. Antenna with ten antennomeres, club with four antennomeres and straight, 1.2 times as long as remaining antennomeres combined. Mentum elevated and slightly flattened anteriorly. Labrum transverse, short, not produced medially, with weak median sinuation.

Pronotum moderately transverse, almost twice as wide as long, widest at base; lateral margins weakly evenly convex and weakly convergent, more strongly convergent in anterior third; anterior angles distinctly produced and sharp; posterior angles strongly rounded; anterior margin with a fine and complete marginal line, weakly convexly produced medially; surface densely and finely punctate, with minute setae in punctures; lateral and anterior border sparsely setose; hypomeron distinctly carinate basally. Scutellum long, with fine, dense punctures, at base punctures less dense, glabrous.

Elytra short-oval, widest in posterior third; striae weakly impressed, finely and moderately densely punctate; even intervals flat, with evenly and moderately dense punctures; odd intervals convex, with sparse, fine punctures concentrated along striae, impunctate medially, with minute setae in punctures. Epipleural edge fine, ending at moderately curved external apical angle of elytra; epipleura densely setose; apical border with a fine rim of microtrichomes (visible at 100× magnification).

Ventral surface dull, finely and densely punctate. Metasternum except long seta on disc nearly glabrous, sparsely covered with minute setae in punctures. Metacoxa glabrous, with a few single setae laterally. Abdominal sternites finely and densely punctuate, glabrous except minute setae in punctures, with a transverse row of coarse punctures each bearing a robust long seta. Mesosternum between mesocoxae as wide as mesofemur. Ratio of length of metepisternum/metacoxa: 1/1.32. Pygidium weakly convex and dull, coarsely and densely punctate, with narrow smooth midline, with a few long setae at apex, otherwise glabrous.

Legs moderately slender. Femora with two longitudinal rows of setae, finely and sparsely punctate. Metafemur moderately shiny and sparsely finely punctate; anterior margin acute, behind anterior margin without serrated line; posterior margin entirely serrated ventrally and moderately widened at apex; posterior margin finely serrated dorsally, glabrous. Metatibia slender and moderately long, widest at apex, ratio of width/length: 1/3.2; dorsal margin finely carinate, with two groups of spines; basal group at middle, apical group at three quarters of metatibial length; in basal half with a few short robust setae in single robust punctures with serrated margin; external face longitudinally convex, finely and sparsely punctate; ventral margin finely serrated, with three robust setae, with the apical one being more distant; medial face impunctate, glabrous, apex shallowly sinuate interiorly near tarsal articulation. Tarsomeres ventrally with sparse, short setae, not carinate laterally, with fine sparse punctures dorsally; metatarsomeres with a strongly serrated ventral ridge; metatarsomere I as long as following two tarsomeres combined and half of its length longer than dorsal tibial spur. Protibia short, bidentate, not widened laterally before basal tooth; anterior claws symmetrical, basal tooth of inner claw sharply truncate at apex.

Aedeagus. Fig. 2E–G.

Female unknown.

#### Diagnosis.

*Neoserica liangi* sp. n. differs form *Neoserica menghaiensis* sp. n. by the shape of the aedeagus: the phallobase is strongly widened apically (in lateral view); the left paramere is much wider at base being evenly narrowed towards the apex rather than being narrowed abruptly after base.

#### Etymology.

The new species is named after the collector of the holotype, Liang Geqiu.

### Neoserica (s. l.) calvoides sp. n.

Taxon classificationAnimaliaColeopteraScarabaeidae

http://zoobank.org/71938A51-1BE2-4B1E-B6CF-46F7A08836AA

Figs 2I–L , 6

#### Type material examined.

Holotype: ♂ [China] “Gulinqing, Maguan, Yunnan, 20.VII.2006, leg. Mao Benyong, Lang Juntong etc.” (HBUM).

#### Description.

Body length: 5.8 mm, length of elytra: 4.6 mm, width: 3.5 mm. Body moderately oblong, dark reddish brown, antennal club yellowish brown, dorsal surface dull and nearly glabrous, labroclypeus and anterior two thirds of frons shiny.

Labroclypeus subtrapezoidal, little wider than long, widest at base; lateral margins strongly convergent and convex anteriorly; anterior angles blunt; anterior margin distinctly sinuate medially; margins moderately reflexed; surface weakly elevated medially and shiny, finely and densely punctate, with a few single setae. Frontoclypeal suture indistinctly incised, weakly elevated and moderately angled medially. Smooth area anterior to eye approximately 3 times as wide as long. Ocular canthus short and narrow, finely and sparsely punctate, with a single terminal seta. Frons on posterior half dull; finely and densely punctate; with a few erect setae beside eyes and dense fine setae on posterior half. Eyes very large, ratio diameter/interocular width: 1.0. Antenna with ten antennomeres, club with four antennomeres and weakly reflexed, 1.4 times as long as remaining antennomeres combined. Mentum elevated and slightly flattened anteriorly. Labrum transverse, short, not produced medially, with weak median sinuation.

Pronotum short and transverse, twice as wide as long, widest shortly before base; lateral margins nearly straight and subparallel, weakly convex and convergent in anterior third; anterior angles weakly produced and blunt; posterior angles blunt, slightly rounded at tip; anterior margin with a fine and complete marginal line, weakly convexly produced medially; surface densely and finely punctate, with minute setae in punctures; lateral and anterior border sparsely setose; hypomeron distinctly carinate basally. Scutellum long, with fine, dense punctures, at base punctures less dense, glabrous.

Elytra oblong, widest at middle; striae weakly impressed, finely and moderately densely punctate; even intervals flat, with evenly and moderately dense punctures; odd intervals convex, with sparse, fine punctures concentrated along striae, impunctate medially, with minute setae in punctures; penultimate lateral interval with a few long single setae. Epipleural edge fine, ending at moderately curved external apical angle of elytra; epipleura densely setose; apical border with a fine rim of microtrichomes (visible at 100× magnification).

Ventral surface dull, finely and densely punctate. Metasternum except long seta on disc nearly glabrous, sparsely covered with minute setae in punctures. Metacoxa glabrous, with a few single setae laterally. Abdominal sternites finely and densely punctuate, glabrous except minute setae in punctures, with a transverse row of coarse punctures each bearing a robust long seta. Mesosternum between mesocoxae as wide as mesofemur. Ratio of length of metepisternum/metacoxa: 1/1.35. Pygidium weakly convex and dull, coarsely and densely punctate, without smooth midline, with a few long setae at apex, otherwise glabrous.

Legs moderately slender. Femora with two longitudinal rows of setae, finely and sparsely punctate. Metafemur moderately shiny and sparsely finely punctate; anterior margin acute, behind anterior margin without serrated line; posterior margin entirely serrated ventrally and moderately widened at apex; posterior margin finely serrated dorsally, glabrous. Metatibia slender and moderately long, widest at apex, ratio of width/length: 1/3.2; dorsal margin moderately carinate, with one group of spines, only; former basal group reduced to a single spine at middle, apical group at three quarters of metatibial length; with a blunt carina beside dorsal margin in basal half bearing a few short robust setae in single robust punctures with serrated margin; external face longitudinally convex, coarsely but sparsely punctate; ventral margin finely serrated, with three robust setae, with the apical one being more distant; medial face impunctate, glabrous, apex moderately truncate interiorly near tarsal articulation. Tarsomeres ventrally with sparse, short setae, not carinate laterally, with fine sparse punctures dorsally; metatarsomeres with a strongly serrated ventral ridge; metatarsomere I as long as following two tarsomeres combined and half of its length longer than dorsal tibial spur. Protibia short, bidentate, not widened laterally before basal tooth; anterior claws symmetrical, basal tooth of inner claw sharply truncate at apex.

Aedeagus. Fig. 2I–K.

Female unknown.

#### Diagnosis.

*Neoserica calvoides* sp. n. differs form *Neoserica calva* by the slightly shorter antennal club and the shape of the parameres: the right paramere bears a strongly widened basal lobe that is curved dorsally; the left paramere is reduced in length and not visible under the basal lobe of the right paramere.

#### Etymology.

The new species is named ‘*calvoides*’ (as combination of *calva*, and – oides (resembling) – the ancient greek suffix (eidos, “form”, “likeness”), referring to the external similarity to *Neoserica calva* (Frey).

### Neoserica (s. l.) gulinqingensis sp. n.

Taxon classificationAnimaliaColeopteraScarabaeidae

http://zoobank.org/E5578439-5EA0-40CF-841D-C7813281E9DD

Figs 2A–D , 6

#### Type material examined.

Holotype: ♂ [China] “Gulinqing, Maguan, Yunnan, 20.VII.2006, leg. Mao Benyong, Lang Juntong etc.” (HBUM).

#### Description.

Body length: 5.9 mm, length of elytra: 4.2 mm, width: 3.2 mm. Body oblong, dark reddish brown, antennal club yellowish brown, dorsal surface dull and nearly glabrous, labroclypeus and anterior two thirds of frons shiny.

Labroclypeus subtrapezoidal, little wider than long, widest at base; lateral margins convergent and convex anteriorly; anterior angles moderately rounded; anterior margin distinctly sinuate medially; margins moderately reflexed; surface weakly elevated medially and shiny, finely and densely punctate, with a few single setae. Frontoclypeal suture indistinctly incised, weakly elevated and moderately angled medially. Smooth area anterior to eye approximately 2.5 times as wide as long. Ocular canthus moderately long and narrow, finely and sparsely punctate, with a single terminal seta. Frons on posterior half dull; finely and densely punctate; with a few erect setae beside eyes and behind frontoclypeal suture, dense fine setae on posterior half. Eyes very large, ratio diameter/interocular width: 0.94. Antenna with ten antennomeres, club with four antennomeres and weakly reflexed, 1.4 times as long as remaining antennomeres combined. Mentum elevated and slightly flattened anteriorly. Labrum transverse, short, not produced medially, with weak median sinuation.

Pronotum transverse, nearly twice as wide as long, widest shortly before base; lateral margins weakly convex and convergent in anterior third, weakly narrowed posteriorly; anterior angles weakly produced and blunt; posterior angles blunt, slightly rounded at tip; anterior margin with a fine and complete marginal line, weakly convexly produced medially; surface densely and finely punctate, with minute setae in punctures; lateral and anterior border sparsely setose; hypomeron distinctly carinate basally. Scutellum long, with fine, dense punctures, at base punctures less dense, glabrous.

Elytra oblong, widest at middle; striae weakly impressed, finely and moderately densely punctate; even intervals flat, with evenly and moderately dense punctures; odd intervals convex, with sparse, fine punctures concentrated along striae, impunctate medially, with minute setae in punctures; penultimate lateral interval with a few long single setae. Epipleural edge fine, ending at moderately curved external apical angle of elytra; epipleura densely setose; apical border with a fine rim of microtrichomes (visible at 100× magnification).

Ventral surface dull, finely and densely punctate. Metasternum except long seta on disc nearly glabrous, sparsely covered with minute setae in punctures. Metacoxa glabrous, with a few single setae laterally. Abdominal sternites finely and densely punctuate, glabrous except minute setae in punctures, with a transverse row of coarse punctures each bearing a robust long seta. Mesosternum between mesocoxae as wide as mesofemur. Ratio of length of metepisternum/metacoxa: 1/1.32. Pygidium weakly convex and dull, coarsely and densely punctate, without smooth midline, with a few long setae at apex, otherwise glabrous.

Legs moderately slender. Femora with two longitudinal rows of setae, finely and sparsely punctate. Metafemur moderately shiny and sparsely finely punctate; anterior margin acute, behind anterior margin without serrated line; posterior margin entirely serrated ventrally and moderately widened at apex; posterior margin finely serrated dorsally, glabrous. Metatibia slender and moderately long, widest at apex, ratio of width/length: 1/3.3; dorsal margin moderately carinate, with one group of spines, only; former basal group reduced to a single spine at middle, apical group at three quarters of metatibial length; with a blunt carina beside dorsal margin in basal half bearing a few short robust setae in single robust punctures with serrated margin; external face longitudinally convex, coarsely but sparsely punctate; ventral margin finely serrated, with three robust setae, with the apical one being more distant; medial face impunctate, glabrous, apex moderately truncate interiorly near tarsal articulation. Tarsomeres ventrally with sparse, short setae, not carinate laterally, with fine sparse punctures dorsally; metatarsomeres with a strongly serrated ventral ridge; metatarsomere I as long as following two tarsomeres combined and half of its length longer than dorsal tibial spur. Protibia short, bidentate, not widened laterally before basal tooth; anterior claws symmetrical, basal tooth of inner claw sharply truncate at apex.

**Figure 3.**
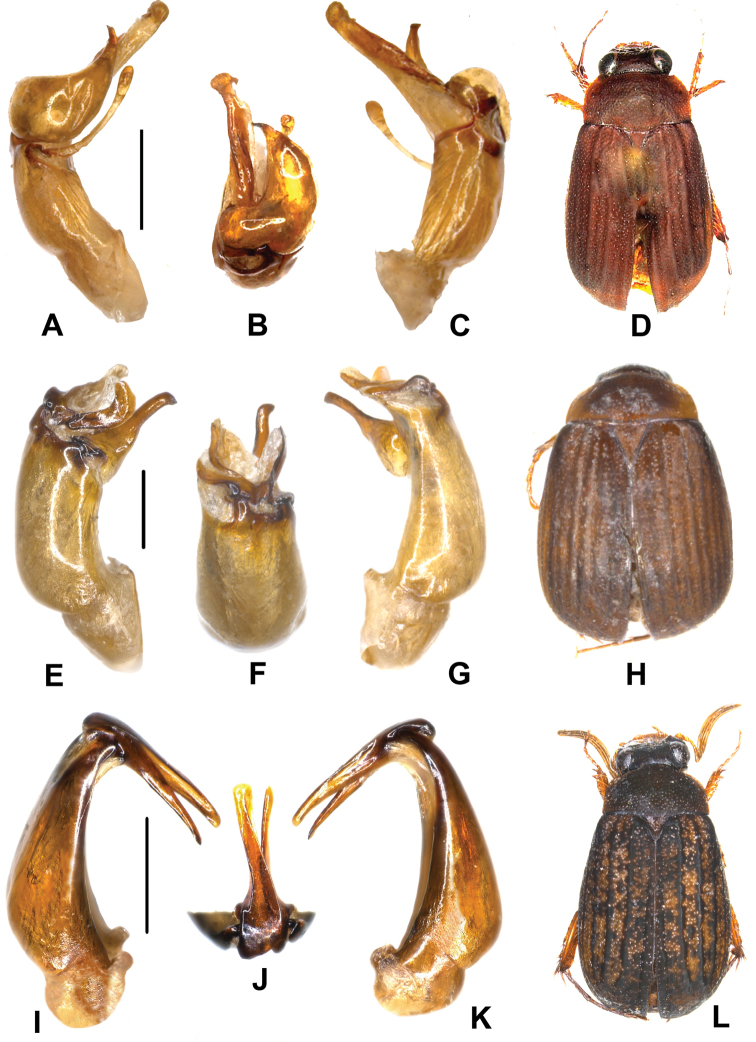
**A–D** *Neoserica gulinqingensis* sp. n. (holotype) **E–H** *Neoserica anonyma* sp. n. (holotype) **I–L** *Neoserica zheijangensis* sp. n. (holotype). **A, E, I** aedeagus, left side lateral view **C, G, K** aedeagus, right side lateral view **B, F, J** parameres, dorsal view **D, H, L** habitus. Scale: 0.5 mm. Habitus not to scale.

Aedeagus. Fig. 3A–C.

Female unknown.

#### Diagnosis.

*Neoserica gulinqingensis* sp. n. differs form *Neoserica calvoides* by the shape of the parameres: the left paramere is not reduced in length, subequal in length to the less widened dorsal lobe of the right paramere.

#### Etymology.

The new species is named after its type locality, Gulinqing.

### Neoserica (s. l.) anonyma sp. n.

Taxon classificationAnimaliaColeopteraScarabaeidae

http://zoobank.org/5596AE75-4B9B-44CB-8C69-71B36A90FC96

Figs 3E–H , 6

#### Type material examined.

Holotype: ♂ “China, W. Yunnan, env. Baoshan, 2500m, 2.–3.viii.2002, leg. S. Murzin, I. Shokhin” (CPPB). Paratypes: 2 ♂♂, 16 ♀♀ “China, W. Yunnan, env. Baoshan, 2500m, 2.–3.viii.2002, leg. S. Murzin, I. Shokhin” (CPPB, ZFMK).

#### Description.

Body length: 5.1 mm, length of elytra: 3.9 mm, width: 3.5 mm. Body oval, dark reddish brown, antennal club yellowish brown, dorsal surface dull and nearly glabrous, labroclypeus and anterior half of frons shiny.

Labroclypeus subtrapezoidal, distinctly wider than long, widest at base; lateral margins strongly convergent and convex anteriorly; anterior angles blunt; anterior margin distinctly sinuate medially, sharply reflexed medially; margins moderately reflexed; surface strongly elevated medially and shiny, finely and densely punctate, with a few single setae. Frontoclypeal suture indistinctly incised, weakly elevated and moderately angled medially. Smooth area anterior to eye narrow, approximately as wide as long. Ocular canthus short and narrow, finely and sparsely punctate, with a single terminal seta. Frons on posterior half dull; finely and densely punctate; with a few erect setae beside eyes and behind frontoclypeal suture, with dense, fine setae on posterior half. Eyes small, ratio diameter/interocular width: 0.58. Antenna with ten antennomeres, club with four antennomeres and straight, as long as remaining antennomeres combined. Mentum elevated and slightly flattened anteriorly. Labrum transverse, short, not produced medially, with weak median sinuation.

Pronotum moderately transverse, widest at base; lateral margins weakly evenly convex and weakly convergent anteriorly; anterior angles distinctly produced and sharp; posterior angles blunt, slightly rounded at tip; anterior margin straight with a very fine and complete marginal line; surface densely and finely punctate, with minute setae in punctures; lateral and anterior border sparsely setose; hypomeron distinctly carinate basally. Scutellum large, with fine, dense punctures, glabrous.

Elytra short-oval, widest shortly behind middle; striae weakly impressed, finely and moderately densely punctate; intervals weakly convex, with moderately dense punctures concentrated along striae, with minute setae in punctures. Epipleural edge fine, ending at moderately curved external apical angle of elytra; epipleura densely setose; apical border with a fine rim of microtrichomes (visible at 100× magnification).

Ventral surface dull, finely and densely punctate. Metasternum except long seta on disc nearly glabrous, sparsely covered with minute setae in punctures. Metacoxa glabrous, with a few single setae laterally. Abdomen missing in the holotype. Mesosternum between mesocoxae as wide as mesofemur. Ratio of length of metepisternum/metacoxa: 1/1.2.

Legs moderately slender. Femora with two longitudinal rows of setae, finely and sparsely punctate. Metafemur moderately shiny and sparsely finely punctate; anterior margin acute, behind anterior margin without serrated line; posterior margin entirely serrated ventrally and moderately widened at apex; posterior margin finely serrated dorsally, glabrous. Metatibia slender and moderately long, widest at apex, ratio of width/length: 1/3.3; dorsal margin distinctly carinate, with two groups of spines; basal group at middle, apical group at three quarters of metatibial length; in basal half with a few short robust setae in single robust punctures with serrated margin; external face longitudinally convex, finely and sparsely punctate; ventral margin finely serrated, with three robust setae, with the apical one being more distant; medial face impunctate, glabrous, apex moderately truncate interiorly near tarsal articulation. Tarsomeres ventrally with sparse, short setae, not carinate laterally, impunctate dorsally; metatarsomeres with a strongly serrated ventral ridge; metatarsomere I slightly shorter than following two tarsomeres combined and nearly half of its length longer than dorsal tibial spur. Protibia short, bidentate, not widened laterally before basal tooth; anterior claws symmetrical, basal tooth of inner claw sharply truncate at apex.

Aedeagus. Fig. 3E–G.

#### Diagnosis.

*Neoserica anonyma* sp. n. differs form all other species of the *Neoserica calva* group with small eyes and short antennal club by the parameres being both subequal in length.

#### Etymology.

This new species was named based on the Latin word “*anonymus*” (anonymous), with reference to its inconspicuous external appearance what made it hard initially to group this species systematically. Therefore type specimens were originally labelled as “*Maladera anonyma*”.

#### Variation.

Body length of the paratypes: 5.1–6.1 mm, length of elytra: 3.9–4.2 mm, width: 3.5–3.6 mm. Female has the antennal club composed of three antennomeres, as long as the remaining antennomeres combined.

#### Remarks.

Abdomen of the holotype was lost during specimens shipment after genitalia already were dissected.

### Neoserica (s. l.) zheijangensis sp. n.

Taxon classificationAnimaliaColeopteraScarabaeidae

http://zoobank.org/15082F5C-3FD3-48FC-93A1-0178651935DA

Figs 3I–L , 6

#### Type material examined.

Holotype: ♂ “China, SW Zheijang, 5.VI. Fangyangshan, Huangmao Jian 27°53'N, 119°11'E, 1500–1850m Jaroslav Turna leg., 2008” (ZFMK). Paratypes: 1 ♂ [China] “Kuatun (2300 m) 27,40 n.Br. 117,40 ö.L. J. Klapperich 19.5. 1938 (Fukien)” (NMPC), 1 ♂ [China] “Longmenhe River, Xingshan, Hubei, 7.V.1994, 1300m, leg. Yao Jian” (IZAS), 1 ♂ [China] “San’gang, Chong’anxingcun, Fujian, 27.V.1960, 740m, leg. Zhang Yiran” (IZAS).

#### Description.

Body length: 5.9 mm, length of elytra: 4.0 mm, width: 3.2 mm. Body oblong, dark reddish brown, labroclypeus and irregular spots on elytra reddish brown, antennal club yellowish brown, dorsal surface dull and nearly glabrous, labroclypeus and anterior half of frons shiny.

Labroclypeus subrectangular, little wider than long, widest at base; lateral margins convex and moderately convergent anteriorly; anterior angles strongly rounded; anterior margin moderately sinuate medially; margins moderately reflexed; surface flat and shiny, coarsely and finely, very densely punctate, with a few single setae. Frontoclypeal suture distinctly incised, weakly elevated and moderately angled medially. Smooth area anterior to eye approximately 1.5 times as wide as long. Ocular canthus moderately long and narrow, finely and sparsely punctate, with a terminal seta. Frons on posterior half dull; coarsely and densely punctate; with a few erect setae beside eyes and behind frontoclypeal suture, otherwise only with minute setae. Eyes large, ratio diameter/interocular width: 0.83. Antenna with ten antennomeres, club with four antennomeres and strongly reflexed, 2.3 times as long as remaining antennomeres combined. Mentum elevated and slightly flattened anteriorly. Labrum transverse, short, not produced medially, with weak median sinuation.

Pronotum short, widest at base; lateral margins nearly straight and subparallel in basal half, moderately convex and strongly convergent anteriorly; anterior angles weakly produced and blunt, slightly rounded at tip; posterior angles nearly right-angled and moderately rounded at tip; anterior margin with a fine and complete marginal line, weakly convexly produced medially; surface densely and finely punctate, with minute setae in punctures; lateral and anterior border sparsely setose; hypomeron distinctly carinate basally. Scutellum long, triangular, with fine, very dense punctures, glabrous, along midline punctures less dense.

Elytra oblong, widest in posterior third; striae weakly impressed, finely and moderately densely punctate; even intervals flat, with evenly and moderately dense punctures; odd intervals convex, with sparse, fine punctures concentrated along striae, impunctate medially, with minute setae in punctures. Epipleural edge fine, ending at moderately curved external apical angle of elytra; epipleura densely setose; apical border with a fine rim of microtrichomes (visible at 100× magnification).

Ventral surface dull, finely and densely punctate. Metasternum except long seta on disc nearly glabrous, sparsely covered with minute setae in punctures. Metacoxa glabrous, with a few single setae laterally. Abdominal sternites finely and densely punctuate, glabrous except minute setae in punctures, with a transverse row of coarse punctures each bearing a robust long seta. Mesosternum between mesocoxae as wide as mesofemur. Ratio of length of metepisternum/metacoxa: 1/1.5. Pygidium weakly convex and dull, coarsely and moderately densely punctate, without smooth midline, with a few long setae at apex, otherwise glabrous.

Legs slender. Femora with two longitudinal rows of setae, finely and sparsely punctate. Metafemur moderately shiny and sparsely finely punctate; anterior margin acute, behind anterior margin without serrated line; posterior margin in apical half serrated ventrally and moderately widened at apex; posterior margin finely serrated dorsally, glabrous. Metatibia slender and moderately long, widest at apex, ratio of width/length: 1/3.4; dorsal margin sharply carinate, with two groups of spines; basal group at middle, apical group at three quarters of metatibial length; basally with a few short robust setae in single robust punctures; external face longitudinally convex, coarsely and densely punctate; ventral margin finely serrated, with three robust setae, with the apical one being slightly more distant; medial face densely and finely punctate, glabrous, apex sharply truncate interiorly near tarsal articulation. Tarsomeres ventrally with sparse, short setae, not carinate laterally, with fine sparse punctures dorsally; metatarsomeres with a strongly serrated ventral ridge, punctures dense and longitudinally impressed dorsally, sharply carinate laterally; metatarsomere I as long as following two tarsomeres combined and half of its length longer than dorsal tibial spur. Protibia moderately long, bidentate, not widened before basal lateral tooth; anterior claws symmetrical, basal tooth of inner claw sharply truncate at apex.

Aedeagus. Fig. 3I–K.

Female unknown.

#### Diagnosis.

*Neoserica zheijangensis* differs from all other species of the *Neoserica calva* group by the bicoloured elytra having reddish spots on the dark background, as well as by the antennal club being composed of five antennomeres.

#### Etymology.

The new species is named after its occurrence in Zheijang Province.

#### Variation.

Body length of the paratypes: 5.1–5.9 mm, length of elytra: 3.9–4.0 mm, width: 2.9–3.2 mm.

### Neoserica (s. l.) zhibenshanica sp. n.

Taxon classificationAnimaliaColeopteraScarabaeidae

http://zoobank.org/1FC196B0-78CA-4200-B1DB-7ACF7CDB4704

Figs 4A–D , 6

#### Type material examined.

Holotype: ♂ [China] “Mts. Zhibenshan, Yunlong, Yunnan, 20.VI.1981, 2500m, leg. Zhang Xuezhong” (IZAS).

#### Description.

Body length: 6.6 mm, length of elytra: 5.0 mm, width: 3.5 mm. Body oblong, dark reddish brown, antennal club yellowish brown, dorsal surface dull and nearly glabrous, labroclypeus and anterior half of frons shiny.

Labroclypeus subrectangular, little wider than long, widest at base; lateral margins straight and subparallel; anterior angles strongly rounded; anterior margin moderately sinuate medially; margins strongly reflexed; surface flat and moderately shiny, coarsely and very densely punctate, with a few single setae. Frontoclypeal suture distinctly incised, weakly elevated and moderately angled medially. Smooth area anterior to eye approximately 1.5 times as wide as long. Ocular canthus long and moderately narrow, finely and densely punctate, with a terminal seta. Frons on posterior half dull; coarsely and densely punctate; with a few erect setae beside eyes and behind frontoclypeal suture, otherwise only with minute setae. Eyes moderately large, ratio diameter/interocular width: 0.64. Antenna with ten antennomeres, club with four antennomeres and strongly reflexed, 3 times as long as remaining antennomeres combined. Mentum elevated and slightly flattened anteriorly. Labrum transverse, short, not produced medially, with weak median sinuation.

Pronotum short, widest at base; lateral margins nearly straight and subparallel in basal half, moderately convex and strongly convergent anteriorly; anterior angles weakly produced and blunt, slightly rounded at tip; posterior angles nearly right-angled and moderately rounded at tip; anterior margin with a fine and complete marginal line, weakly convexly produced medially; surface densely and finely punctate, with minute setae in punctures; lateral and anterior border sparsely setose; hypomeron distinctly carinate basally. Scutellum long, triangular, with fine, very dense punctures, glabrous, along midline punctures less dense.

Elytra oblong, widest in posterior third; striae weakly impressed, finely and moderately densely punctate; even intervals flat, with evenly and moderately dense punctures; odd intervals convex, with sparse, fine punctures concentrated along striae, impunctate medially, with minute setae in punctures. Epipleural edge fine, ending at moderately curved external apical angle of elytra; epipleura densely setose; apical border with a fine rim of microtrichomes (visible at 100× magnification).

Ventral surface dull, finely and densely punctate. Metasternum except long seta on disc nearly glabrous, sparsely covered with minute setae in punctures. Metacoxa glabrous, with a few single setae laterally. Abdominal sternites finely and densely punctuate, glabrous except minute setae in punctures, with a transverse row of coarse punctures each bearing a robust long seta. Mesosternum between mesocoxae as wide as mesofemur. Ratio of length of metepisternum/metacoxa: 1/1.24. Pygidium moderately convex and dull, coarsely and moderately densely punctate, without smooth midline, with a few long setae at apex, otherwise glabrous.

Legs slender. Femora with two longitudinal rows of setae, finely and sparsely punctate. Metafemur moderately shiny and sparsely finely punctate; anterior margin acute, behind anterior margin without serrated line; posterior margin in apical half serrated ventrally and moderately widened at apex; posterior margin finely serrated dorsally, glabrous. Metatibia slender and long, widest at apex, ratio of width/length: 1/4.2; dorsal margin sharply carinate, with two groups of spines; basal group at middle, apical group at three quarters of metatibial length; in basal half with a blunt carina beside dorsal margin bearing a few single short and robust setae in robust punctures with serrated margin; external face longitudinally convex, coarsely and moderately densely punctate; ventral margin finely serrated, with three robust equidistant setae; medial face densely and finely punctate, glabrous, apex sharply truncate interiorly near tarsal articulation. Tarsomeres ventrally with sparse, short setae, not carinate laterally, without punctures dorsally; metatarsomeres with a strongly serrated ventral ridge; metatarsomere I distinctly shorter than following two tarsomeres combined and nearly half of its length longer than dorsal tibial spur. Protibia moderately long, bidentate, not widened before basal lateral tooth; anterior claws symmetrical, basal tooth of inner claw sharply truncate at apex.

**Figure 4.**
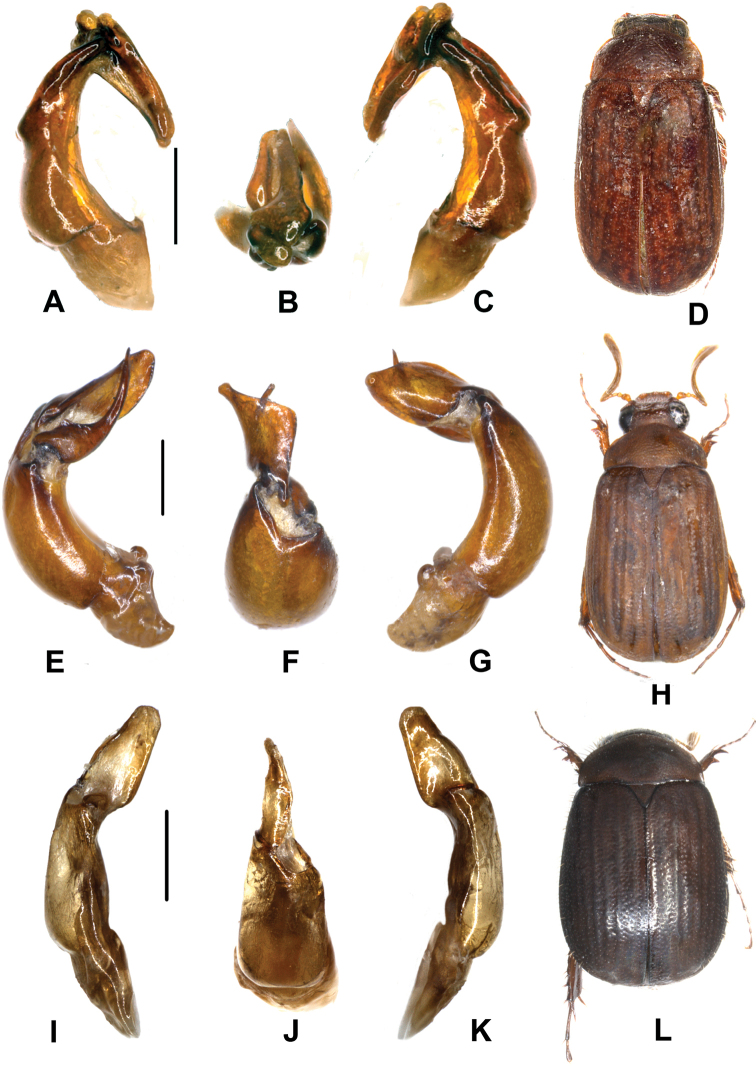
**A–D** *Neoserica zhibenshanica* sp. n. (holotype) **E–H** *Neoserica taipingensis* sp. n. (holotype) **I–L** *Neoserica mengi* sp. n. (holotype). **A, E, I** aedeagus, left side lateral view **C, G, K** aedeagus, right side lateral view **B, F, J** parameres, dorsal view **D, H, L** habitus. Scale: 0.5 mm. Habitus not to scale.

Aedeagus. Fig. 4A–C.

Female unknown.

#### Diagnosis.

*Neoserica zhibenshanica* sp. n. differs from all other species of the *Neoserica calva* group with smaller eyes by the length of the antennal club being 3 times as long as the remaining antennomeres combined.

#### Etymology.

The new species is named after its type locality, Mt. Zhibenshan.

### Neoserica (s. l.) taipingensis sp. n.

Taxon classificationAnimaliaColeopteraScarabaeidae

http://zoobank.org/35C780C3-6489-4D0D-986A-EC2360F43B4F

Figs 4E–H , 6

#### Type material examined.

Holotype: ♂ “China-Shaanxi, SW Tsinling Mts., Taiping vill., 33°33'N, 106°43'E, June 2000, 1500–2000m, Siniaev & Plutenko leg.” (CPPB). Paratypes: 12 ♂♂, 1 ♀ “China-Shaanxi, SW Tsinling Mts., Taiping vill., 33°33'N, 106°43'E, June 2000, 1500–2000m, Siniaev & Plutenko leg.” (CPPB, ZFMK), 2 ♂♂ “China, Shaanxi, Tsingling Mts., 1600m, Nat. Res. Foping, 33°51'N, 107°57'E, 20.iv.–11.v.1999, V. Siniaev & A. Plutenko lgt.” (CPPB), 1 ♂ “China, Shaanxi, Panda area, Nat. Res. Foping, 1600m, 6–11.iv.1999, 33°45'N, 107°48'E, V. Siniaev & A. Plutenko lgt.” (CPPB), 1 ♂ [China] “Meixian County, Shaanxi, VIII.1963, leg. Chen You’guang” (IZAS).

#### Description.

Body length: 7.2 mm, length of elytra: 4.8 mm, width: 3.7 mm. Body oblong, reddish brown, antennal club yellowish brown, dorsal surface dull and nearly glabrous, labroclypeus and anterior two thirds of frons shiny.

Labroclypeus subrectangular, little wider than long, widest at base; lateral margins straight and subparallel in basal half, strongly convergent and convex anteriorly; anterior angles moderately rounded; anterior margin moderately sinuate medially; margins moderately reflexed; surface flat and shiny, coarsely and densely punctate, with a few single setae. Frontoclypeal suture distinctly incised, weakly elevated and moderately angled medially. Smooth area anterior to eye approximately 1.5 times as wide as long. Ocular canthus long and narrow, finely and densely punctate, with a short terminal seta. Frons on posterior third dull; coarsely and densely punctate; with a few erect setae beside eyes and behind frontoclypeal suture, otherwise only with minute setae. Eyes large, ratio diameter/interocular width: 0.85. Antenna with ten antennomeres, club with four antennomeres and strongly reflexed, 2.5 times as long as remaining antennomeres combined. Mentum elevated and slightly flattened anteriorly. Labrum transverse, short, not produced medially, with weak median sinuation.

Pronotum short, widest shortly behind middle; lateral margins evenly moderately convex and convergent anteriorly and posteriorly; anterior angles weakly produced and blunt, slightly rounded at tip; posterior angles blunt and rounded at tip; anterior margin with a fine and complete marginal line, weakly convexly produced medially; surface densely and finely punctate, with minute setae in punctures; lateral and anterior border sparsely setose; hypomeron distinctly carinate basally. Scutellum long, triangular, with fine, very dense punctures, glabrous.

Elytra oblong, widest shortly behind middle; striae weakly impressed, finely and moderately densely punctate; even intervals flat, with evenly and moderately dense punctures; odd intervals convex, with sparse, fine punctures concentrated along striae, impunctate medially, with minute setae in punctures. Epipleural edge fine, ending at moderately curved external apical angle of elytra; epipleura densely setose; apical border with a fine rim of microtrichomes (visible at 100× magnification).

Ventral surface dull, finely and densely punctate. Metasternum except long seta on disc nearly glabrous, sparsely covered with minute setae in punctures. Metacoxa glabrous, with a few single setae laterally. Abdominal sternites finely and densely punctuate, glabrous except minute setae in punctures, with a transverse row of coarse punctures each bearing a robust long seta. Mesosternum between mesocoxae as wide as mesofemur. Ratio of length of metepisternum/metacoxa: 1/1.22. Pygidium strongly convex and dull, coarsely and densely punctate, without smooth midline, with a few long setae.

Legs slender. Femora with two longitudinal rows of setae, finely and sparsely punctate. Metafemur moderately shiny and sparsely finely punctate; anterior margin acute, behind anterior margin without serrated line; posterior margin in apical half serrated ventrally and moderately widened at apex; posterior margin finely serrated dorsally, glabrous. Metatibia slender and moderately long, widest at apex, ratio of width/length: 1/3.4; dorsal margin sharply carinate, with two groups of spines; basal group at middle, apical group at three quarters of metatibial length; in basal half with a blunt carina beside dorsal margin bearing a few single short and robust setae in robust punctures with serrated margin; external face longitudinally convex, coarsely and moderately densely punctate; ventral margin finely serrated, with two robust widely distant setae; medial face sparsely and finely punctate, glabrous, apex sharply truncate interiorly near tarsal articulation. Tarsomeres ventrally with sparse, short setae, without punctures dorsally; metatarsomeres with a strongly serrated ventral ridge, weakly carinate laterally; metatarsomere I distinctly shorter than following two tarsomeres combined and nearly half of its length longer than dorsal tibial spur. Protibia moderately long, bidentate, not widened before basal lateral tooth; anterior claws symmetrical, basal tooth of inner claw sharply truncate at apex.

Aedeagus. Fig. 4E–G.

#### Diagnosis.

*Neoserica taipingensis* differs from *Neoserica calva* by the smaller eyes, slightly longer antennal club as well as by the shape of the parameres: the right paramere is shell-like widened and nearly straight.

#### Etymology.

The new species is named after its type locality, Taiping.

#### Variation.

Body length of the paratypes: 6.6–7.8 mm, length of elytra: 4.7–5.2 mm, width: 3.7–4.1 mm. Female has the antennal club composed of three antennomeres, as long as the remaining antennomeres combined.

### Neoserica (s. l.) mengi sp. n.

Taxon classificationAnimaliaColeopteraScarabaeidae

http://zoobank.org/89ECCAD8-A385-4E44-893A-CCDE78E28212

Figs 4I–L , 6

#### Type material examined.

Holotype: ♂ “X-DA2984 China S. Yunnan (Xishuangbanna) 23km NW Jinghong vic. Na Ban (NNNR), 730m, 22°09.49'N, 100°39.92'E 20.x.2008 L. Meng Neoserica spCHz1 ♂” (ZFMK). Paratypes: 1 ♂ [China] “Mt. Wuyanling, Taishun, Zhejiang, 28.VII–3.VIII.2005, leg. Ba Yibin” (HBUM), 4 ♂♂, 1 ♀ [China] “Caiyanghe, Pu’er, Yunnan, 28.VII.2007, leg. Ren Guodong, Hou Wenjun, Li Yalin” (HBUM), 1 ♂ [China] “Caiyanghe, Pu’er, Yunnan, 28.VII.2007, 1700m, leg. Mao Benyong, Xu Jishan” (HBUM), 1 ♂ [China] “Defu, Napo, Guangxi, 15.VIII.1998, 1300m, leg. Huang Fusheng, Li Wenzhu” (IZAS), 1 ♂ [China] “Yunnan, Mt. Fofangshan 2010-7-27/ LW-1056” (ZFMK), 1 ♂ [China] “Yunnan, Mt. Fofangshan 2010-7-27/ LW-1056bis” (ZFMK), 1 ♂ [China] “Yunnan, Nabanhe Nature Reserve, 2008-X-11/ LW-1364” (ZFMK), 2 ♂♂, 1 ♀ [China] “Yexianggu, Jinghong, Yunnan, 3–4.VIII.2006, 850m, leg. Mao Benyong etc.” (HBUM).

#### Description.

Body length: 5.3 mm, length of elytra: 3.9 mm, width: 3.5 mm. Body oval, dark reddish brown, antennal club yellowish brown, dorsal surface dull and nearly glabrous, labroclypeus and anterior half of frons shiny.

Labroclypeus subtrapezoidal, distinctly wider than long, widest at base; lateral margins strongly convergent and convex anteriorly; anterior angles blunt; anterior margin distinctly sinuate medially, sharply reflexed medially; margins moderately reflexed; surface slightly elevated medially and shiny, finely and densely punctate, with a few single setae. Frontoclypeal suture indistinctly incised, weakly elevated and moderately angled medially. Smooth area anterior to eye narrow, approximately as wide as long. Ocular canthus short and narrow, impunctate, with a single terminal seta. Frons on posterior half dull; finely and densely punctate; with a few erect setae beside eyes and behind frontoclypeal suture, with dense, fine setae on posterior half. Eyes small, ratio diameter/interocular width: 0.57. Antenna with ten antennomeres, club with four antennomeres and straight, 1.2 times as long as remaining antennomeres combined. Mentum elevated and slightly flattened anteriorly. Labrum transverse, short, not produced medially, with median sinuation.

Pronotum moderately transverse, widest at base; lateral margins weakly evenly convex and weakly convergent anteriorly; anterior angles distinctly produced and sharp; posterior angles blunt, broadly rounded at tip; anterior margin straight with a very fine and complete marginal line; surface densely and finely punctate, with minute setae in punctures; lateral and anterior border sparsely setose; hypomeron distinctly carinate basally. Scutellum large, with fine, dense punctures, glabrous, punctures on base less dense.

Elytra short-oval, widest shortly behind middle; striae weakly impressed, finely and moderately densely punctate; intervals weakly convex, with moderately dense punctures concentrated along striae, with minute setae in punctures, penultimate lateral interval with a few single, erect setae. Epipleural edge fine, ending at moderately curved external apical angle of elytra; epipleura densely setose; apical border with a fine rim of microtrichomes (visible at 100× magnification).

Ventral surface dull, finely and densely punctate. Metasternum except long seta on disc nearly glabrous, sparsely covered with minute setae in punctures. Metacoxa glabrous, with a few single setae laterally. Abdominal sternites finely and densely punctuate, glabrous except minute setae in punctures, with a transverse row of coarse punctures each bearing a robust long seta. Mesosternum between mesocoxae as wide as mesofemur. Ratio of length of metepisternum/metacoxa: 1/1.33. Pygidium weakly convex and dull, coarsely and densely punctate, without smooth midline, with short adpressed on disc and sides and a few long setae near apex.

Legs moderately slender. Femora with two longitudinal rows of setae, finely and sparsely punctate. Metafemur dull and sparsely finely punctate; anterior margin acute, behind anterior margin without serrated line; posterior margin entirely serrated ventrally and moderately widened at apex; posterior margin finely serrated dorsally, glabrous. Metatibia slender and moderately long, widest at apex, ratio of width/length: 1/2.8; dorsal margin distinctly carinate, with two groups of spines; basal group shortly behind middle, apical group at three quarters of metatibial length; in basal half with a few short robust setae in single robust punctures with serrated margin; external face longitudinally convex, finely and sparsely punctate; ventral margin finely serrated, with three robust setae, with the apical one being more distant; medial face impunctate, glabrous, apex moderately truncate interiorly near tarsal articulation. Tarsomeres ventrally with sparse, short setae, not carinate laterally, impunctate dorsally; metatarsomeres with a strongly serrated ventral ridge; metatarsomere I slightly shorter than following two tarsomeres combined and nearly half of its length longer than dorsal tibial spur. Protibia short, bidentate, not widened laterally before basal tooth; anterior claws symmetrical, basal tooth of inner claw sharply truncate at apex.

Aedeagus. Fig. 4I–K.

#### Diagnosis.

*Neoserica mengi* sp. n. is similar to *Neoserica anonyma* sp. n. externally but differs from it by the slightly longer antennal club, slightly stouter metatibia, and the shape of the parameres: the left paramere is strongly reduced in size, its length is 1/7 of that of the right paramere.

#### Etymology.

This new species is named after one of the collectors of the type series, L. Meng.

#### Variation.

Body length of the paratypes: 5.2–5.9 mm, length of elytra: 3.8–4.0 mm, width: 3.4–3.6 mm. Female has the antennal club composed of three antennomeres, as long as the remaining antennomeres combined.

### Neoserica (s. l.) koelkebecki sp. n.

Taxon classificationAnimaliaColeopteraScarabaeidae

http://zoobank.org/B6C0FA89-FD9D-41EF-AF9B-9F1716818C17

Figs 5A–D , 6

#### Type material examined.

Holotype: ♂ “08.07.2010 Mudeungsan, Gwangju (Südkorea) leg. T. Kölkebeck” (ZFMK).

#### Description.

Body length: 5.8 mm, length of elytra: 4.0 mm, width: 3.2 mm. Body oval, dark reddish brown, antennal club yellowish brown, dorsal surface dull and nearly glabrous, labroclypeus and anterior half of frons shiny.

Labroclypeus subtrapezoidal, distinctly wider than long, widest at base; lateral margins nearly straight and strongly convergent anteriorly; anterior angles weakly rounded; anterior margin distinctly sinuate medially, sharply reflexed medially; margins moderately reflexed; surface slightly elevated medially and shiny, finely and moderately densely punctate, with a few single setae. Frontoclypeal suture invisible. Smooth area anterior to eye narrow, approximately 1.2 times as wide as long. Ocular canthus short and narrow, sparsely punctate, with a single terminal seta. Frons on posterior half dull; finely and sparsely punctate; with a few erect setae beside eyes and behind frontoclypeal suture, with dense, fine setae on posterior half. Eyes small, ratio diameter/interocular width: 0.52. Antenna with ten antennomeres, club with four antennomeres and straight, 1.7 times as long as remaining antennomeres combined. Mentum elevated and slightly flattened anteriorly. Labrum transverse, short, not produced medially, with median sinuation.

Pronotum moderately transverse, widest at base; lateral margins subparallel in basal half, strongly convex at middle, and weakly evenly convex and convergent anteriorly; anterior angles distinctly produced and sharp; posterior angles blunt, broadly rounded at tip; anterior margin nearly straight, with a fine and complete marginal line; surface moderately densely and finely punctate, with minute setae in punctures; lateral and anterior border sparsely setose; hypomeron distinctly carinate basally. Scutellum large, with fine, dense punctures, glabrous.

Elytra short-oval, widest shortly behind middle; striae weakly impressed, finely and moderately densely punctate; intervals weakly convex, with moderately dense punctures concentrated along striae, with minute setae in punctures, penultimate lateral interval with a few single, erect setae. Epipleural edge fine, ending at moderately curved external apical angle of elytra; epipleura densely setose; apical border with a fine rim of microtrichomes (visible at 100× magnification).

Ventral surface dull, finely and densely punctate. Metasternum except long seta on disc nearly glabrous, sparsely covered with minute setae in punctures. Metacoxa glabrous, with a few single setae laterally. Abdominal sternites finely and densely punctuate, glabrous except minute setae in punctures, with a transverse row of coarse punctures each bearing a robust long seta. Mesosternum between mesocoxae as wide as mesofemur. Ratio of length of metepisternum/metacoxa: 1/1.58. Pygidium strongly convex and dull, coarsely and densely punctate, without smooth midline, with short adpressed on disc and sides and a few long setae near apex.

Legs moderately slender. Femora with two longitudinal rows of setae, finely and sparsely punctate. Metafemur shiny and sparsely finely punctate; anterior margin acute, behind anterior margin without serrated line; posterior margin entirely serrated ventrally and moderately widened at apex; posterior margin finely serrated dorsally, glabrous. Metatibia slender and moderately long, widest at apex, ratio of width/length: 1/3.3; dorsal margin indistinctly carinate, with two groups of spines; basal group at middle, apical group at three quarters of metatibial length; in basal half with a few short robust setae in single robust punctures with serrated margin; external face longitudinally convex, finely and sparsely punctate; ventral margin finely serrated, with three robust setae, with the apical one being more distant; medial face impunctate, glabrous, apex moderately truncate interiorly near tarsal articulation. Tarsomeres ventrally with sparse, short setae, not carinate laterally, impunctate dorsally; metatarsomeres with a strongly serrated ventral ridge and a few fine punctures dorsally; metatarsomere I as long as following two tarsomeres combined and nearly half of its length longer than dorsal tibial spur. Protibia short, bidentate, slightly widened laterally before basal tooth; anterior claws symmetrical, basal tooth of inner claw sharply truncate at apex.

**Figure 5.**
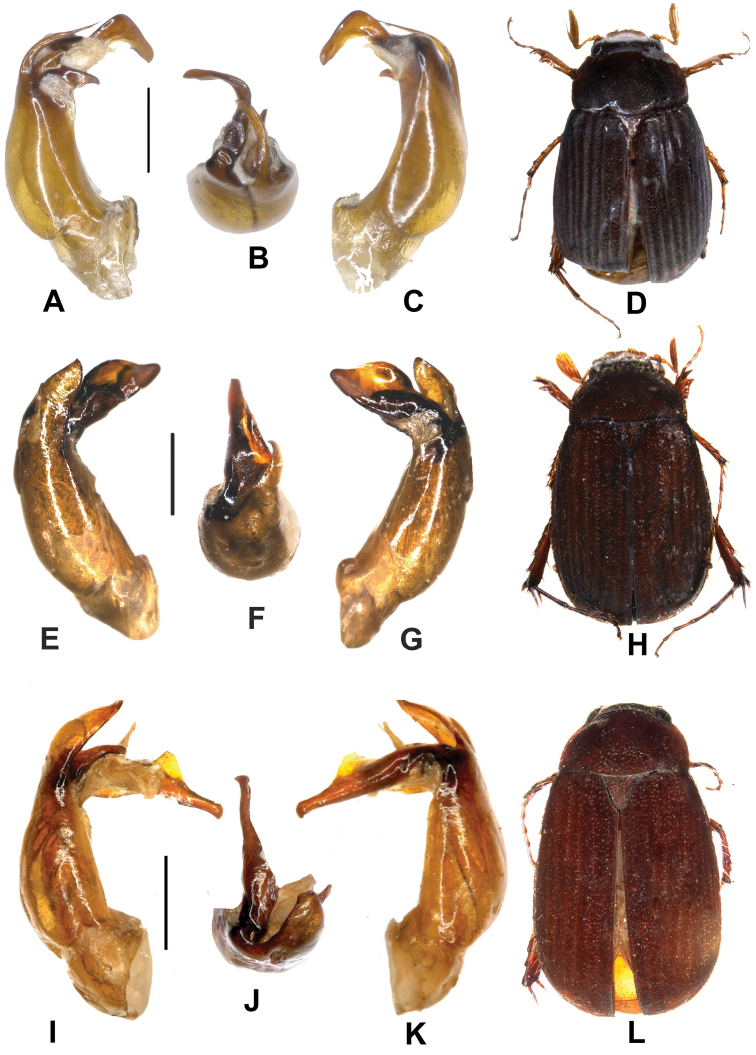
**A–D** *Neoserica koelkebecki* sp. n. (holotype) **E–H** *Neoserica luxiensis* sp. n. (holotype) **I–L** *Neoserica ailaoshanica* sp. n. (holotype). **A, E, I** aedeagus, left side lateral view **C, G, K** aedeagus, right side lateral view **B, F, J** parameres, dorsal view **D, H, L** habitus. Scale: 0.5 mm. Habitus not to scale.

Aedeagus. Fig. 5A–C.

Female unknown.

#### Diagnosis.

*Neoserica koelkebecki* sp. n. is rather similar to *Neoserica anonyma* sp. n. and *Neoserica mengi* sp. n. externally but differs from them by the slightly longer antennal club and the shape of the aedeagus: the apex of the dorsal phallobase has a narrow process.

#### Etymology.

This new species is named after the collector of the species, Torben Kölkebeck.

### Neoserica (s. l.) luxiensis sp. n.

Taxon classificationAnimaliaColeopteraScarabaeidae

http://zoobank.org/5EA97BA7-B353-4E95-AF9F-EC366F161FA4

Figs 5E–H , 6

#### Type material examined.

Holotype: ♂ [China] “China (Yunnan) Dehong Dai Aut. Pref., mount. Range 31km E Luxi, 2280m, 24°29'31"N/ 98°52'58"E (grassland/ pasture, under stones/ shrubs, in moss/ litter) 3.VI.2007 D.W. Wrase [19]” (ZFMK).

#### Description.

Body length: 6.1 mm, length of elytra: 4.6 mm, width: 3.2 mm. Body oblong-oval, dark reddish brown, antennal club yellowish brown, dorsal surface dull and nearly glabrous, labroclypeus and anterior half of frons shiny.

Labroclypeus shortly subtrapezoidal, distinctly wider than long, widest at base; lateral margins nearly straight and convergent anteriorly; anterior angles strongly rounded; anterior margin distinctly sinuate medially; margins moderately reflexed; surface slightly elevated medially and shiny, finely and densely punctate, with a few single setae. Frontoclypeal suture invisible. Smooth area anterior to eye narrow, approximately 1.2 times as wide as long. Ocular canthus short and narrow, sparsely punctate, with a single terminal seta. Frons on posterior half dull; finely and moderately densely punctate; with a few long and erect setae beside eyes and behind frontoclypeal suture, with dense, fine setae on posterior half. Eyes small, ratio diameter/interocular width: 0.59. Antenna with ten antennomeres, club with four antennomeres and straight, 1.4 times as long as remaining antennomeres combined. Mentum elevated and slightly flattened anteriorly. Labrum transverse, short, not produced medially, with median sinuation.

Pronotum moderately transverse, widest at base; lateral margins evenly convex and convergent anteriorly; anterior angles distinctly produced and sharp; posterior angles blunt, moderately rounded at tip; anterior margin nearly straight, with a fine and complete marginal line; surface moderately densely and finely punctate, with minute setae in punctures; lateral and anterior border sparsely setose; hypomeron distinctly carinate basally. Scutellum large, with fine, dense punctures, glabrous, on midline punctures less dense.

Elytra short-oval, widest shortly behind middle; striae weakly impressed, finely and moderately densely punctate; intervals weakly convex, with moderately dense punctures concentrated along striae, with minute setae in punctures, lateral odd intervals with a few single, erect setae. Epipleural edge fine, ending at moderately curved external apical angle of elytra; epipleura densely setose; apical border with a fine rim of microtrichomes (visible at 100× magnification).

Ventral surface dull, finely and densely punctate. Metasternum except long seta on disc nearly glabrous, sparsely covered with minute setae in punctures. Metacoxa glabrous, with a few single setae laterally. Abdominal sternites finely and densely punctuate, glabrous except minute setae in punctures, with a transverse row of coarse punctures each bearing a robust long seta. Mesosternum between mesocoxae as wide as mesofemur. Ratio of length of metepisternum/metacoxa: 1/1.39. Pygidium weakly convex and dull, coarsely and densely punctate, without smooth midline, with a few long setae near apex.

Legs moderately slender. Femora with two longitudinal rows of setae, finely and sparsely punctate. Metafemur dull, sparsely and finely punctate; anterior margin acute, behind anterior margin without serrated line; posterior margin entirely serrated ventrally and moderately widened at apex; posterior margin finely serrated dorsally, glabrous. Metatibia slender and moderately long, widest at apex, ratio of width/length: 1/3.5; dorsal margin finely carinate, with two groups of spines; basal group at middle, apical group at three quarters of metatibial length; in basal half with a few short robust setae in single robust punctures with serrated margin; external face longitudinally convex, finely and densely punctate, glabrous; ventral margin finely serrated, with three robust equidistant setae; medial face densely and finely punctate, glabrous, apex moderately truncate interiorly near tarsal articulation. Tarsomeres ventrally with sparse, short setae, not carinate laterally, impunctate dorsally; metatarsomeres with a strongly serrated ventral ridge and a few fine punctures dorsally; metatarsomere I as long as following two tarsomeres combined and nearly half of its length longer than dorsal tibial spur. Protibia short, bidentate, not widened before basal tooth; anterior claws symmetrical, basal tooth of inner claw sharply truncate at apex.

Aedeagus. Fig. 5E–G.

Female unknown.

#### Diagnosis.

*Neoserica luxiensis* sp. n. is rather similar to *Neoserica koelkebecki* sp. n. in general appearance and the shape of the male genitalia. It differs from the latter by the slightly shorter antennal club and the shape of the aedeagus: the process of the apical phallobase is situated dorsolaterally, the right paramere is much wider than that in *Neoserica koelkebecki*.

#### Etymology.

This new species is named after its type locality, Luxi.

### Neoserica (s. l.) ailaoshanica sp. n.

Taxon classificationAnimaliaColeopteraScarabaeidae

http://zoobank.org/97B5900D-3FFE-4107-9623-AA79F5D270F2

Figs 5I–L , 6

#### Type material examined.

Holotype: ♂ “Mts. Ailaoshan, Jingdong, Yunnan, 7–9.VIII.2009, 2450m, leg. Xu Jishan, Zhang Liuxiang etc.” (HBUM).

#### Description.

Body length: 6.5 mm, length of elytra: 4.8 mm, width: 3.8 mm. Body oblong, dark reddish brown, antennal club yellowish brown, dorsal surface dull and nearly glabrous, labroclypeus and anterior half of frons shiny.

Labroclypeus subtrapezoidal, widest at base; lateral margins convex and convergent anteriorly; anterior angles moderately rounded; anterior margin distinctly sinuate medially; margins moderately reflexed; surface slightly elevated medially and shiny, finely and densely punctate, with a few single setae. Frontoclypeal suture indistinctly incised and slightly elevated. Smooth area anterior to eye approximately 1.5 times as wide as long. Ocular canthus moderately long and narrow, sparsely punctate, with a single terminal seta. Frons on posterior half dull; finely and sparsely punctate, anterior midline slightly elevated; with a few long and erect setae beside eyes and behind frontoclypeal suture, with dense, fine setae on posterior half. Eyes small, ratio diameter/interocular width: 0.59. Antenna with ten antennomeres, club with four antennomeres and straight, 1.6 times as long as remaining antennomeres combined. Mentum elevated and slightly flattened anteriorly. Labrum transverse, short, not produced medially, with median sinuation.

Pronotum widest at base; lateral margins evenly convex and convergent anteriorly; anterior angles distinctly produced and sharp; posterior angles blunt, moderately rounded at tip; anterior margin nearly straight, with a fine and complete marginal line; surface moderately densely and finely punctate, with minute setae in punctures; lateral and anterior border sparsely setose; hypomeron distinctly carinate basally. Scutellum large, with fine, very dense punctures, glabrous, on midline impunctate.

Elytra short-oval, widest shortly behind middle; striae weakly impressed, finely and moderately densely punctate; intervals weakly convex, with moderately dense punctures concentrated along striae, with minute setae in punctures, lateral odd intervals with a few single, erect setae. Epipleural edge fine, ending at moderately curved external apical angle of elytra; epipleura densely setose; apical border with a fine rim of microtrichomes (visible at 100× magnification).

Ventral surface dull, finely and densely punctate. Metasternum, except long seta on disc, nearly glabrous, sparsely covered with minute setae in punctures. Metacoxa glabrous, with a few single setae laterally. Abdominal sternites finely and densely punctuate, glabrous except minute setae in punctures, with a transverse row of coarse punctures each bearing a robust long seta. Mesosternum between mesocoxae as wide as mesofemur. Ratio of length of metepisternum/metacoxa: 1/1.38. Pygidium weakly convex and dull, coarsely and densely punctate, without smooth midline, with a few long setae near apex.

Legs moderately slender. Femora with two longitudinal rows of setae, finely and sparsely punctate. Metafemur dull, sparsely and finely punctate; anterior margin acute, behind anterior margin without serrated line; posterior margin entirely serrated ventrally and moderately widened at apex; posterior margin finely serrated dorsally, glabrous. Metatibia slender and moderately long, widest at apex, ratio of width/length: 1/3.7; dorsal margin finely carinate, with two groups of spines; basal group shortly behind middle, apical group at three quarters of metatibial length; in basal half with a few short robust setae in single robust punctures with serrated margin; external face longitudinally convex, finely and densely punctate, glabrous; ventral margin finely serrated, with three robust setae, distal one more distant; medial face densely and finely punctate, glabrous, apex moderately truncate interiorly near tarsal articulation. Tarsomeres ventrally with sparse, short setae, not carinate laterally, impunctate dorsally; metatarsomeres with a strongly serrated ventral ridge and a few fine punctures dorsally; metatarsomere I as long as following two tarsomeres combined and nearly half of its length longer than dorsal tibial spur. Protibia short, bidentate, not widened before basal tooth; anterior claws symmetrical, basal tooth of inner claw sharply truncate at apex.

Aedeagus. Fig. 5I–K.

Female unknown.

#### Diagnosis.

*Neoserica ailaoshanica* sp. n. is rather similar to *Neoserica luxiensis* sp. n. in general appearance and the shape of the male genitalia but differs from it by the slightly longer antennal club and the shape of the aedeagus: the process of the apical phallobase is slightly longer and the right paramere is longer and narrower than that in *Neoserica luxiensis*.

#### Etymology.

The new species is named after its type locality, Ailaoshan.

**Figure 6.**
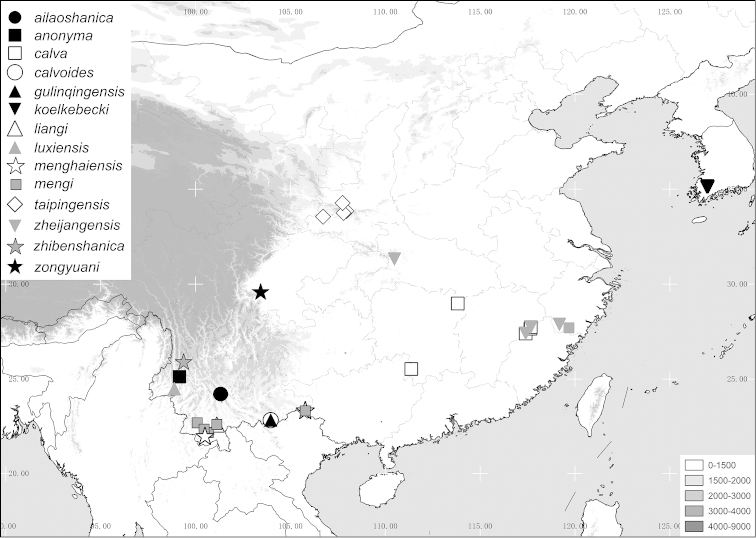
Distribution of the species of the *Neoserica calva* group.

## Supplementary Material

XML Treatment for Neoserica (s. l.) calva

XML Treatment for Neoserica (s. l.) zongyuani

XML Treatment for Neoserica (s. l.) menghaiensis

XML Treatment for Neoserica (s. l.) liangi

XML Treatment for Neoserica (s. l.) calvoides

XML Treatment for Neoserica (s. l.) gulinqingensis

XML Treatment for Neoserica (s. l.) anonyma

XML Treatment for Neoserica (s. l.) zheijangensis

XML Treatment for Neoserica (s. l.) zhibenshanica

XML Treatment for Neoserica (s. l.) taipingensis

XML Treatment for Neoserica (s. l.) mengi

XML Treatment for Neoserica (s. l.) koelkebecki

XML Treatment for Neoserica (s. l.) luxiensis

XML Treatment for Neoserica (s. l.) ailaoshanica
